# N^6^‐Methyladenosine Modification on the Function of Female Reproductive Development and Related Diseases

**DOI:** 10.1002/iid3.70089

**Published:** 2024-12-11

**Authors:** Xiangrong Cui, Huihui Li, Xia Huang, Tingting Xue, Shu Wang, Xinyu Zhu, Xuan Jing

**Affiliations:** ^1^ Reproductive Medicine Center The affiliated Children's Hospital of Shanxi Medical University, Children's Hospital of Shanxi, Shanxi Maternal and Child Health Hospital Taiyuan China; ^2^ Department of Clinical Laboratory Shanxi Provincial People's Hospital, Shanxi Medical University Taiyuan China

**Keywords:** epigenetic modification, female reproductive development, N6‐methyladenosine (m6A), oocyte maturation, reproductive diseases

## Abstract

**Background:**

N6‐methyladenosine (m6A) modification is a widespread and reversible epigenetic alteration in eukaryotic mRNA, playing a pivotal role in various biological functions. Its significance in female reproductive development and associated diseases has recently become a focal point of research.

**Objective:**

This review aims to consolidate current knowledge of the role of m6A modification in female reproductive tissues, emphasizing its regulatory dynamics, functional significance, and implications in reproductive health and disease.

**Methods:**

A comprehensive analysis of recent studies focusing on m6A modification in ovarian development, oocyte maturation, embryo development, and the pathogenesis of reproductive diseases.

**Results:**

m6A modification exhibits dynamic regulation in female reproductive tissues, influencing key developmental stages and processes. It plays critical roles in ovarian development, oocyte maturation, and embryo development, underpinning essential aspects of reproductive health. m6A modification is intricately involved in the pathogenesis of several reproductive diseases, including polycystic ovary syndrome (PCOS), premature ovarian failure (POF), and endometriosis, offering insights into potential molecular mechanisms and therapeutic targets.

**Conclusion:**

The review highlights the crucial role of m6A modification in female reproductive development and related diseases. It underscores the need for further research to explore innovative diagnostic and therapeutic strategies for reproductive disorders, leveraging the insights gained from understanding m6A modification's impact on reproductive health.

## Graphical abstract

N6‐methyladenosine (m6A) modification, a prevalent and reversible epigenetic modification in eukaryotic mRNA, has emerged as a crucial regulator in various biological processes. Recent studies have shed light on the significant role of m6A modification in female reproductive development and its related diseases. This review aims to summarize the current understanding of m6A modification in female reproductive tissues, including its dynamic regulation and functional importance in ovarian development, oocyte maturation, and embryo development. Furthermore, we discuss the involvement of m6A modification in the pathogenesis of reproductive diseases such as polycystic ovary syndrome (PCOS), premature ovarian failure (POF), and endometriosis, highlighting potential molecular mechanisms and therapeutic targets. By providing a comprehensive overview of m6A modification in female reproductive health, this review underscores the importance of further research in this field to develop innovative strategies for diagnosis and treatment of reproductive disorders.

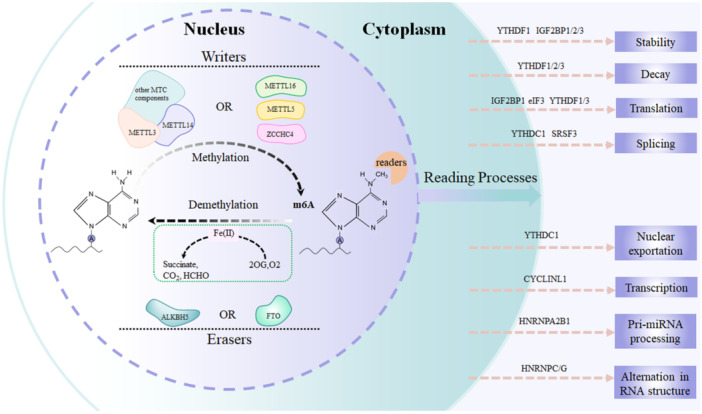

## 1 Introduction

Recent studies have highlighted the significant role of epigenetic modifications, including DNA methylation, histone modification, chromatin rearrangement, and RNA modifications, in regulating various physiological and pathological processes. These processes encompass embryonic development [1], stem cell differentiation [2] and tumorigenesis, thereby attracting growing attention in the field of bioscience research [1]. While the modification of DNA and proteins has been extensively investigated, RNA modification still presents a significant research challenge [3]. To date, more than 170 types of chemical modifications have been identified, collectively referred to as the epitranscriptome [4]. Among the most crucial types in humans are N6‐methyladenosine (m^6^A), C5‐methylcytidine (m^5^C), C3‐methylcytidine (m^3^C), N1‐methyladenosine (m^1^A), 7‐methylguanosine (m^7^G), and N6, 2’‐O‐dimethyladenosine (m^6^Am), as presented in Table 1.

**Table 1:** List of RNA methylation characteristics of m^6^A, m^5^C, m^3^C, m^1^A, m^7^G, and m^6^Am methylation.

RNA methylations	Modification sites	Transmethylases	Demethyltransferases	Reading proteins	Distribution	References
m^6^A	the 6th position nitrogen atom of adenine	METTL3/14/16, WTAP, RBM15, ZC3H13, VIRMA, CBLL1	FTO, ALKBH5	YTHDF1/2/3, YTHDC1/2, IGF2BP1/2/3, HNRNPA2B1, HNRNPC	mRNA, circRNA, miRNA, lncRNA	[5, 6, 7, 8, 9, 10, 11, 12, 13]
m^6^Am	the 6th position nitrogen atom of adenine	PCIF1, METTL3/4	FTO		snRNA, mRNA	[14, 15, 16]
m^1^A	the 1st position nitrogen atom of adenine	TRMT6/61A/61B/10C	ALKBH1, ALKBH3	YTHDF1/2/3, YTHDC1	tRNA, rRNA, mRNA	[17]
m^3^C	the 3rd position nitrogen atom of cytimidine	TbTRM140a, METTL2A/2B/6/8	ALKBH1, ALKBH3		mRNA, tRNA	[18, 19]
m^5^C	the 5th position nitrogen atom of cytimidine	NSUN1‐7, TRDMT1, DNMT2	TET1/2, ALK‐BH1	FMRP, ALYREF, YBX1	rRNA, tRNA, mRNA, enhancer RNA	[20]
m^7^G	the 5th position nitrogen atom of guanine	METTL1, WBSCR22, WDR4, TRMT112			mRNA, tRNA, rRNA, miRNA	[21]

Among these various modification types, N6‐methyladenosine (m^6^A) stands out as a highly prevalent internal mRNA modification in eukaryotes. Initially identified through studies on the nucleotide composition of poly(A) RNA in 1974 [22], m6A accounts for over 80% of all RNA methylation modifications [23]. It is predominantly located near the stop codon and plays a crucial role in regulating a multitude of cellular processes, such as RNA stability, metabolism, splicing, and translation [4, 24]. In mammals, approximately 0.1%‐0.6% of adenines undergo m^6^A modification, with an average of 3–5 methylation sites per mRNA. Importantly, m^6^A modifications can be selectively deposited onto transcripts in tissue‐ and cell type‐specific patterns. Various methods, including analytical chemistry, high‐throughput sequencing, M6A‐CLIP, miCLIP, and SELECT‐m^6^A, have been developed for the detection of m^6^A. These techniques enable the identification of specific methylation sites and the quantification of the modification fraction at these sites, thereby facilitating biological investigations of RNA modification [25]. This technological advancement offers a valuable approach for exploring the roles of m^6^A‐modified proteins in physiological and pathological processes. Recent studies have highlighted the significant role of m6A modification in the female reproductive system, with its dysregulation closely linked to oocyte maturation [26], premature ovarian insufficiency (POI) [27] and other reproductive system disorders [28, 29]. Despite these findings, the specific functions and precise mechanisms of m^6^A modification in this context remain incompletely understood. This review aims to summarize recent research advancements regarding the impact of m^6^A modification on female reproductive function. It delves into the underlying molecular mechanisms and detection methodologies to offer comprehensive insights for a better comprehension of female reproductive system disorders.

## 2 The Mechanism and Regulation of m^6^A RNA Methylation

**Figure 1:**
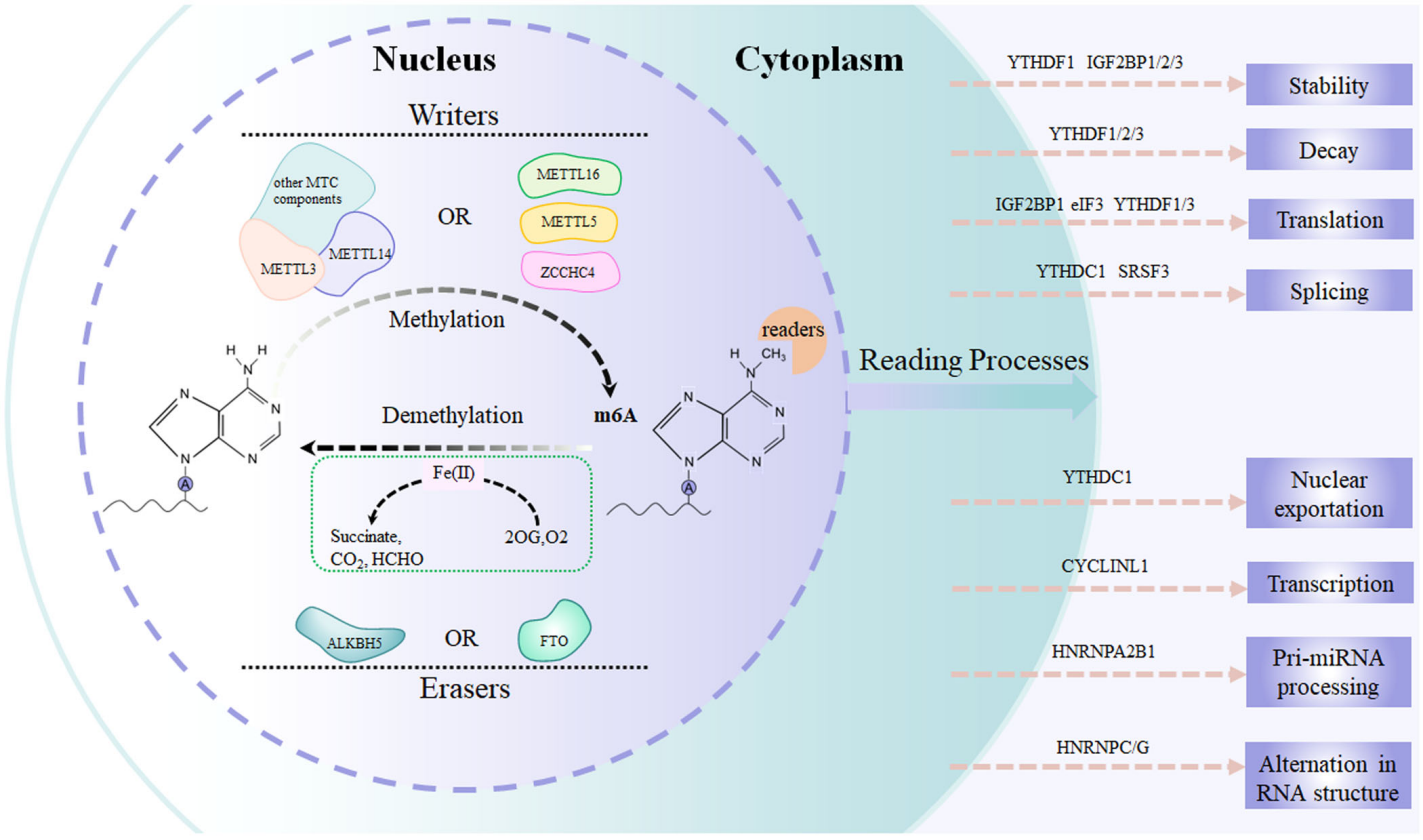
Overview of the reversible m^6^A RNA modifications and related functions. This figure presents a comprehensive diagram illustrating the dynamic and reversible process of m^6^A RNA modification, highlighting the key enzymes involved in the methylation (writers), demethylation (erasers), and the recognition (readers) of these modifications. Additionally, it outlines the pivotal roles these modifications play in regulating various RNA metabolic processes, such as splicing, export, stability, and translation, which are crucial for cellular function and homeostasis. Through this figure, we aim to convey the complexity and significance of m^6^A modifications in RNA biology and their overarching implications in gene expression regulation.

m^6^A modification refers to the methylation of the 6th nitrogen atom of RNA adenylate, making it the most prevalent RNA epigenetic modification found in eukaryotic mRNAs [30]. In mammals, the typical mRNA harbors approximately 3–5 m^6^A modifications, with the m^6^A to adenine ratio ranging from 0.1% to 0.4% [31]. m^6^A sites, characterized by a consensus motif of 5′‐RRACH‐3′ (where *R* = *A*/*G* and *H* = *A*/*C*/*U*), are situated in evolutionarily conserved regions of the human and mouse transcriptomes. These m^6^A sites are predominantly located within internal mRNA sequences, frequently in proximity to stop codons, particularly in the 3′ untranslated regions (UTRs) of mRNAs encoded by various genes [32]. Similar to DNA methylation, m^6^A modification is a dynamic and reversible process that is rigorously regulated by methyltransferases (writers), demethylases (erasers), and m^6^A‐binding proteins (readers) [33] (Figure 1 and Table 1).

### 2.1 Methyltransferase (m^6^A Writers)

The “write in” of m^6^A modifications is catalyzed by the N^6^‐adenosine methyltransferase complex (MTC), which includes methyltransferase‐like 3/14/16 (METTL3/14/16), Wilms' tumor 1‐associated protein (WTAP), RNA‐binding motif proteins 15 (RBM15), virlike m^6^A methyltransferase associated protein (VIRMA/KIAA1429), Cbl proto‐oncogene like 1 (CBLL1), and zinc finger CCCH‐type containing 13 (ZC3H13). These components facilitate the transfer of methyl groups from S‐adenosylmethionine (SAM) to RNA adenosine for methylation. METTL3, the first identified m6A methyltransferase, serves as the sole catalytic subunit of MTC and forms stable heterodimers regulated by WTAP. METTL14, a molecular homolog of METTL3, plays a crucial role in enhancing RNA substrate recognition and binding by establishing a stable complex with METTL3. While METTL14 is not directly involved in catalysis, it aids in the specific binding of RNA substrates and stabilizes the N6‐adenosine MTC to enhance catalytic activity. Depletion of either METTL3 or METTL14 can markedly decrease the level of m6A modification across the entire transcriptome, consequently affecting mRNA stability and resulting in cellular dysfunction [47]. Numerous studies have underscored the significance of METTL3/14 in gonad development and oocyte maturation. Loss of expression of METTL3/14 can lead to gonadal or gamete dysplasia, ultimately causing impaired fertility [48, 49, 50].

As a regulatory subunit, WTAP lacks obvious catalytic domains but functions in recruiting the stable heterodimer core complex of METTL3/14 to nuclear speckles (associated with mRNA export). This recruitment is mediated by amino acids 5–13 of the nuclear localization signal (NLS) (‐PLPKKVRL‐ to ‐PLPGGVGL‐) located at the N‐terminus of WTAP, thereby enhancing me–thylation efficiency [28]. Interestingly, WTAP‐dependent methylation has been observed to exhibit an inverse relationship with mRNA stability and tends to be depleted from highly transcribed messages. This enrichment may have evolved to prevent mRNA methylation, thereby optimizing their stability [51]. RBM15 interacts with WTAP to facilitate the recruitment of the N6‐adenosine MTC to its target sites, a process crucial for regulating m^6^A‐mediated X‐chromosome inactivation in humans [5, 52]. VIRMA/KIAA1429 is considered a scaffold for MTC, aiding in the deposition of m6A in the 3′ UTR region and stop codon of target mRNA [6, 53]. CBLL1, also known as Hakai, is a conserved component of MTC essential for stabilizing the m^6^A mRNA methylation machinery [54]. Within the nucleus, ZC3H13 serves as an anchor for WTAP, VIRMA/KIAA1429, and Hakai, retaining the MTC in nuclear speckles through its LC domain and facilitating m^6^A methylation [55].

METTL16, a recently identified methyltransferase, is predominantly found in 3′UTRs. Deletion of METTL16 has been shown to lead to a decrease in m^6^A levels by a minimum of 20% [7]. Accumulated studies have demonstrated that METTL16 plays roles in gene regulation through both methyltransferase activity‐dependent and ‐independent mechanisms [7, 56]. Within the cell nucleus, METTL16 functions as an m^6^A writer, catalyzing the deposition of m6A into hundreds of specific mRNA targets [56]. In the cytosol, METTL16 enhances translation in an m^6^A‐independent manner [56]. While METTL16 is capable of catalyzing the formation of m^6^A in RNA by utilizing SAM as a methyl donor, it is predominantly found within introns or at intron‐exon boundaries, which distinguishes it from the typical m^6^A sites located in UTRs [57]. Moreover, METTL16 exhibits a preference for methylating double‐stranded RNA structures that harbor a nonameric consensus sequence (UACAGAGAA, with the underlined A representing the modified adenosine), whereas METTL3/14 shows a preference for single‐stranded RNAs containing a short and degenerate motif (DRACH, where *D* = *A*/*G*/U; *R* = *A*/*G*; *H* = *A*/*C*/*U*; with the underlined A indicating the modified adenosine) [57]. In particular, cytosolic METTL16 plays a role in promoting the formation of the translation initiation complex (TIC) and the translation of more than 4000 mRNA transcripts by interacting with eukaryotic translation initiation factors and ribosomal RNAs (rRNAs) [58, 59].

### 2.2 Demethylases (m^6^A Erasers)

Demethylases function as erasers, reversing m^6^A methylation with its dynamic and reversible characteristics. This process involves active demethylation by m^6^A demethylases in a manner dependent on ferrous iron and α‐ketoglutaric acid [60, 61]. Demethylases, in conjunction with m^6^A methyltransferase, regulate the m^6^A levels of transcripts, thereby influencing the downstream effects of m^6^A readers. Identified demethylases to date include fat mass and obesity‐associated (FTO) and α‐ketoglutarate‐dependent dioxygenase alkB homolog 5 (ALKH5), both of which are members of the ALKb family of dioxygenases known for their ability to demethylate N‐methylated nucleic acids [60, 61].

FTO, the first demethylase identified in 2011, is predominantly found in nuclear speckles where it removes m^6^A residues from RNA. It partially co‐localizes with the splicing factor SART1 or other splicing‐related factors [60]. In humans, FTO is a gene spanning approximately 400 kb with eight introns and nine exons situated on chromosome 16q12.2. FTO catalyzes the oxidative demethylation of 4‐methylthymine and 3‐methyluracil in single‐stranded DNA and RNA [60, 62]. FTO has the ability to sequentially oxidize m^6^A methylation to the intermediate N^6^‐hydroxymethyladenosine (hm^6^A) and N6‐formyladenosine (f^6^A), which are metastable and can subsequently be hydrolyzed to adenine [62]. Studies have shown that FTO‐mediated N6‐methyladenosine plays a role in regulating spermatogenesis in an age‐dependent manner [63]. Intriguingly, recent research has revealed that FTO is functionally significant in mouse oocyte and embryonic development. It achieves this by mediating m^6^A demethylation to modulate the RNA abundance of long‐interspersed element‐1 (LINE1) and influencing the local chromatin state [64].

ALKBH5, another crucial m^6^A demethylase, contains an alanine‐rich region at the N‐terminus and a distinctive coiled‐coil structure. This enzyme catalyzes the direct removal of m^6^A modifications in the nucleus [65]. The active site of ALKBH5 is more exposed compared to FTO, potentially due to FTO having a longer N‐terminal region and C‐terminal domain. These structural elements serve to obstruct the active site in FTO [66]. Studies have indicated that ALKBH5 is essential for regulating the splicing and stability of mRNA during germ cell development and spermatogenesis [67, 68]. Additionally, ALKBH15 and METTL14 are expressed not only in spermatogenic cells but also in stromal cells. Both enzymes collectively participate in the regulation of testosterone synthesis in an m^6^A‐dependent manner [69]. In addition to its involvement in male infertility, ALKBH5 plays a crucial role in determining oocyte quality by regulating the clearance of meiosis‐coupled mRNA in oocytes through m^6^A demethylation [65].

### 2.3 m^6^A‐Binding Proteins (m^6^A Readers)

A growing number of m^6^A‐binding proteins, known as “readers,” recognize RNA methylation modifications to perform their biological functions, resulting in diverse biological phenotypes. Readers function through two reading modes: direct reading and indirect reading. Key proteins involved in direct reading, which selectively bind to the RNA m^6^A region, include YT521‐B homology (YTH) domain‐containing proteins, insulin‐like growth factor 2 mRNA‐binding proteins (IGF2BPs), and eukaryotic initiation factor 3 (eIF3) [70]. On the other hand, heterogeneous nuclear ribonucleoproteins (HNRNPs) are indirect reading proteins that primarily modulate the secondary structure of RNA through m^6^A modifications [71]. The human genome encodes five YTH domain proteins, consisting of three YTHDF domain proteins (YTHDF1, YTHDF2, and YTHDF3) and two YTHDC domain proteins (YTHDC1 and YTHDC2), all of which serve as authentic m^6^A “readers” [70, 72]. Moreover, YTHDF1/2/3 predominantly identify and attach to the RNA m^6^A site in the cytoplasm, whereas YTHDC1/2 function within the nucleus [72]. Among them, YTHDF2 is the initial human YTH domain‐containing protein discovered to interact with over 3000 cellular RNAs via the conserved core motif of Gm^6^AC [73]. YTHDF2 destabilizes and accelerates the degradation of target m^6^A‐containing RNAs by directly recruiting the CCR4‐NOT deadenylase complex in mammalian cells [74]. YTHDF1 interacts with initiation factors to enhance translation efficiency, thereby ensuring efficient protein synthesis from m^6^A‐containing RNAs [75]. Similar to the function of YTHDF2, YTHDF3 controls the deacetylation of target mRNAs by recruiting the PAN2‐PAN3 deacetylase complex, thereby influencing their subcellular localization and alternative splicing [76]. YTHDC1 is a nuclear m^6^A reader protein that modulates the splicing of target mRNAs by recruiting and regulating pre‐mRNA splicing factors [77]. YTHDC2 is a presumed RNA helicase that increases translational activity or promotes mRNA decay by recognizing m^6^A through its YTH domain [78]. Unlike YTHDFs, IGF2BPs promote mRNA stability and storage in an m^6^A‐dependent manner to increase the abundance of their mRNA targets. Conversely, silencing IGF2BPs can result in a global downregulation of gene expression output [79]. Moreover, eIF3 directly interacts with a single m^6^A site in the 5′UTR and initiates translation in an eIF4E‐independent manner [80]. Members of the HNRNP family proteins, such as HNRNPC, HNRNPG, and HNRNPA2B1, are predominantly located in the nucleus. The m^6^A reader proteins HNRNPC and HNRNPG exhibit a preference for binding to m^6^A‐modified RNAs through a mechanism known as the “m^6^A switch,” where m^6^A methylation induces structural changes in the mRNA, destabilizing the RNA hairpin structure and exposing a single‐stranded binding motif for HNRNPC or HNRNPG, facilitating processing. In contrast, HNRNPA2B1 recognizes its m^6^A‐modified targets by directly binding to the methylated RGAC consensus site to regulate RNA splicing and processing [81]. Interaction between HNRNPA2B1 and m^6^A mRNA, facilitated by the interaction with the DGCR8 protein, enhances the biogenesis of microRNAs by recruiting the microprocessor complex (Drosha and Pasha) to the primary miRNAs (pri‐miRNAs) [81, 82].

## 3 Biological Function of m^6^A Modification

At the transcript level, m^6^A, as the most prevalent and evolutionarily conserved base modification, plays a crucial role in regulating almost all aspects of RNA metabolism, encompassing mRNA stability, splicing, localization, nuclear export, and translation. Through thermodynamic mechanisms, m^6^A methylation can alter the secondary structure of RNA, thereby modulating the accessibility of interactions between RNA motifs and RNA‐binding proteins (RBPs). This phenomenon is commonly referred to as m^6^A switching [22]. Figure 1 illustrates the typical regulatory effects of m6A and its functional components on the mRNA life cycle.

### 3.1 m^6^A in mRNA Splicing

Recent discoveries suggest that mRNAs containing poly(A) tails aggregate in distinct nuclear regions known as nuclear polyadenylation domains (NPADs) throughout oocyte development. These NPADs are rich in RNA‐binding proteins and are involved in governing the splicing, maturation, storage, and degradation of the precursor mRNAs [83]. Prior investigations have demonstrated that m^6^A is more prevalent in pre‐mRNA compared to mature mRNA, with m^6^A sites primarily clustered in the intronic region. As a result, the m^6^A modification has been widely acknowledged as a mechanism for modulating splicing. Studies have revealed that METTL3 depletion generally promotes exon skipping and intron retention events [69]. The m^6^A modification of the RNA guanylyltransferase and 5′‐phosphatase (RNGTT) 3′UTR by METTL3 modifies the RNA capping of a limited number of mRNAs, some of which coincide with 5′ terminal oligopyrimidine (TOP) mRNAs and cytoplasmic capped mRNAs [84]. m^6^A methylation is also associated with alternative polyadenylation (APA). Deletion of m^6^A writers can trigger APA events, with the majority of m^6^A sites in the final exon of the gene situated in the 3′UTR region where APA sites are located. Recent investigations on mRNA polyadenosine tails have shown that m^6^A‐modified transcript variants tend to utilize nearby APA sites, leading to a reduced 3′UTR length [36].

### 3.2 m^6^A in mRNA Transport

To promote translation, mature mRNAs need to be transported from the nucleus to the cytoplasm in conjunction with the orderly assembly of the transcription export (TREX) complex on the mRNA and subsequent recruitment of the nuclear heterodimeric export receptor NXF1‐P15. The m^6^A modification is also implicated in this process, modulating gene expression levels by influencing the nuclear export of mRNA. Depletion of the m^6^A writers METTL3, WTAP/KIAA1429, or YTHDC1 has been observed to impede the nuclear export of mRNA associated with the TREX Complex [85, 86]. As an adaptor in the NXF1‐p15 pathway, knockdown of RBM15 leads to a defect in mRNA export [85, 87]. Conversely, depletion of the m^6^A erasing protein ALKBH5 has been shown to increase the cytoplasmic accumulation of polyA^+^ RNA [87]. Further investigations have demonstrated that the m^6^A reader protein YTHDC1 is also involved in regulating the nuclear export of mRNA. Facilitated by the nuclear protein YTHDC1, methylated mRNA interacts with splicing factors and nuclear export adaptors before being transported to nuclear export receptors, thereby enhancing its nuclear export efficiency [87, 88]. These findings highlight a close relationship between m^6^A methylation and pre‐mRNA processing and maturation.

### 3.3 m^6^A in mRNA Translation

Most m^6^A sites are situated in the coding regions of mRNAs. This modification has the ability to influence the decoding of codons by aminoacyl‐tRNA (aa‐tRNA) at the A site of the ribosome [89]. In the process of protein synthesis, the modification of an m^6^A site can impact the interaction between aa‐tRNA and the corresponding codon, thus potentially altering the precision or efficacy of protein translation [90]. Investigating the role of m^6^A in translational regulation involves analyzing the specificity of modification sites and targets, as well as understanding the collaboration or opposition of various proteins under specific conditions.

The mRNA translation process, which allows for swift alterations in the proteome without requiring de novo transcription, is pivotal in enabling prompt responses to various environmental stimuli. Evidence indicates that m6A modification governs translation efficiency by promoting the interaction between reading proteins and translation factors. YTHDF1 specifically binds to m^6^A‐modified mRNAs, leading to the recruitment of eIF3, which in turn triggers ribosome attachment to mRNAs, ultimately boosting translation efficiency [91]. Conversely, the elongation of the 3′UTR length could lead to a rise in miRNA binding sites, consequently impeding the translation process [91]. Furthermore, METTL3 facilitates protein translation from mRNAs in an A cap‐independent manner through the recognition of m^6^A in the 5′UTR or 3′UTR region. In the process of translation initiation, it recruits eIF3, thereby augmenting the translation of particular eIF4E‐dependent mRNAs [92]. In conclusion, m^6^A governs mRNA translation through various mechanisms, with m^6^A modifications in diverse RNA functional regions commonly displaying unique functions.

### 3.4 m^6^A in mRNA Stability

Degradation represents the ultimate stage in mRNA metabolism, wherein the mRNA structure progressively becomes unstable and ultimately undergoes degradation. The potential correlation between m^6^A modification and the control of RNA stability was first explored in 1978 [93]. The researchers proposed that the swift elimination of m^6^A in the cytoplasm could be linked to the short half‐life of mRNAs containing high m^6^A content, indicating that mRNAs with elevated m^6^A levels are more prone to degradation. This hypothesis has since been substantiated, with the knockout of METTL3, leading to a substantial reduction in m^6^A levels in mRNAs, significantly prolonging their half‐life [94]. METTL3 regulates TFRC ubiquitination and ferroptosis by stabilizing NEDD4L mRNA, thereby influencing stroke outcomes [95]. m^6^A modification can modulate mRNA decay through various molecular mechanisms, primarily facilitated by m^6^A reader proteins. YTHDF2 has been identified as the key player in the m^6^A‐dependent mRNA decay pathway, providing the initial direct evidence. Knockdown of YTHDF2 has been shown to enhance the stability of its target mRNA and impede the degradation process [96]. YTHDF2 predominantly recognizes the stop codon region, the 3′UTR, and the coding region (CDS), indicating its potential involvement in mRNA stability and/or translation [96].

The m^6^A reader IGF2BP1 has been shown to reduce the stability of RPL36 and cell proliferation, thereby mediating benzene hematotoxicity by recognizing m^6^A modification [97]. FOXD3‐mediated transactivation of ALKBH5 has been found to promote neuropathic pain through m^6^A‐dependent stabilization of 5‐HT3A mRNA in sensory neurons [98]. Apart from hastening mRNA degradation, m^6^A modification also exerts a more intricate regulatory influence on mRNA stability. For example, the m^6^A reader protein IGF2BPs can bind to RNA stabilizers like HuR, MATR3, and PABPC1. This binding interaction counteracts YTHDF2‐induced decay, thereby enhancing mRNA stability. Additionally, knockdown of methyltransferases METTL3 and METTL14 has been shown to lead to the upregulation of their target mRNA [99].

### 3.5 m^6^A in Noncoding RNA (ncRNA)

Numerous m^6^A modification sites have been identified in ncRNAs, encompassing circular RNAs (circRNAs), long noncoding RNAs (lncRNAs), and microRNAs (miRNAs). m^6^A plays a pivotal role in regulating the cleavage, localization, trafficking, stability, and degradation of ncRNAs, as well as in interacting with ncRNAs to influence cellular biological functions. The interplay among these ncRNAs offers new insights into exploring the underlying mechanisms involved in female reproductive development and associated diseases.

The maturation of miRNA involves the recognition and cleavage of pri‐miRNA primary transcripts by the RNA‐binding protein DGCR8 and the type III ribonuclease Drosha [100]. METTL3 catalyzes the methylation of pri‐miRNA, facilitating its recognition and processing by DGCR8 [101]. Knockdown of METTL3 results in diminished binding of DGCR8 to pri‐miRNA, leading to reduced expression of mature miRNA and accumulation of unprocessed pri‐miRNA [100]. This investigation proposes that m^6^A methylation serves as a novel regulatory mechanism in miRNA processing and represents a significant RNA modification strategy that can modulate the activation of downstream DGCR8 and Drosha, thereby initiating the transformation of pri‐miRNA into mature miRNA.

m^6^A methylation has the capacity to modify the local structure of lncRNAs, facilitating their interaction with the RNA‐binding protein HNRNPC, regulating lncRNA stability, and engaging in various biological processes [102]. Research has demonstrated that m^6^A methylation can influence the localization and function of MALAT1 within the nucleus, modulating its associations with relevant proteins through alterations in RNA structure [103, 104]. Specifically, m^6^A methylation targets the “hairpin” structural region of MALAT1, disrupting U‐A base pairing within this region and diminishing the stability of the MALAT1 “hairpin” structure. This modification promotes the binding of MALAT1 with the RNA‐binding protein HNRNPC, facilitating HNRNPC nuclear export during the G2/M phase of the cell cycle. Consequently, this process enhances the translation of the protein kinase P58, which is crucial for centrosome maturation and bipolar mitotic spindle formation during mitosis, thereby promoting cell division and regulating cell cycle progression [104].

## 4 m^6^A Modification in Female Reproductive Physiology

**Figure 2:**
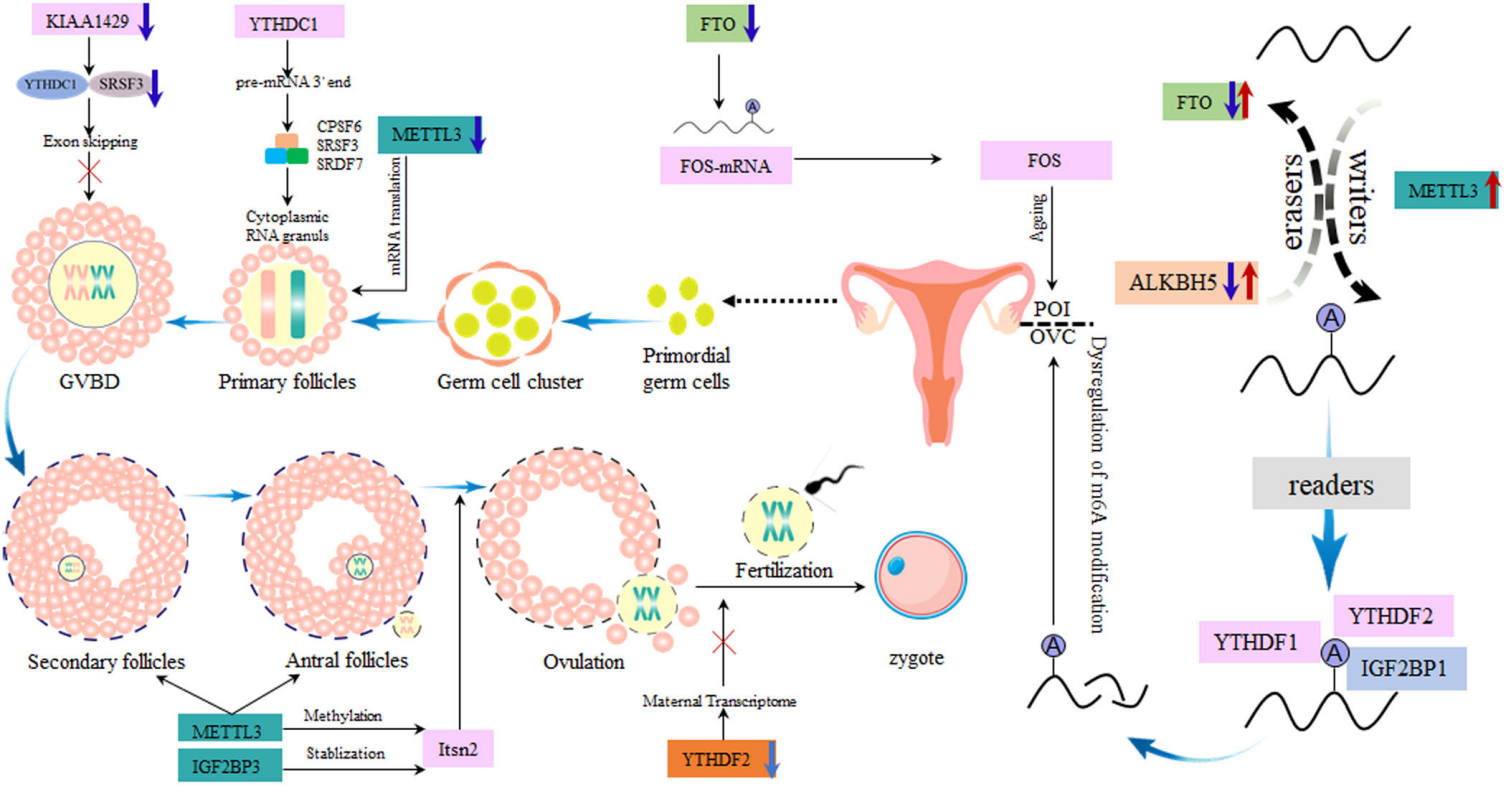
Role of m^6^A on the female reproductive system. In this figure, we delve into the specific impacts of m^6^A modifications on the female reproductive system, illustrating how these modifications influence key aspects of reproductive biology, including ovarian function, oocyte maturation, and embryo development. The figure synthesizes current research findings to depict the multifaceted role of m^6^A in modulating gene expression patterns critical for the healthy development and function of the female reproductive system. Through detailed schematics, we highlight the pathways and mechanisms through which m^6^A modifications exert their effects, underscoring their importance in reproductive health and disease.

The reproductive system is primarily responsible for generating offspring and ensuring the transmission of genetic material from one generation to the next [105]. It relies on the intricate functions of male and female reproductive organs, including processes such as spermatogenesis, follicle development, gametogenesis, embryonic formation, and development [105]. Numerous studies have validated that m^6^A modification governs the differentiation, development, aging, and pathological processes of tissues and organs [106]. Extensive evidence indicates the crucial involvement of m^6^A modification in female reproductive physiology [106], although the precise regulatory mechanisms remain incompletely elucidated. Figure 2 illustrates the regulatory impact of m^6^A modification on the female reproductive process.

### 4.1 Development and Aging of the Female Reproductive System

The female reproductive system comprises the gonads, specifically the ovaries, and the reproductive tract, which includes the uterus, cervix, fallopian tubes, vagina, and external genitals. Its primary function is to produce oocytes and facilitate their transport to the fallopian tubes and uterus for fertilization and embryonic development. The development of the female reproductive system initiates from 5 weeks post‐fertilization and continues until 20 weeks of gestation [107]. The primary sex cord (PSC) of the gonadal ridge originates from the urogenital ridge [108]. During the 5th and 6th week of pregnancy, primordial germ cells (PGCs) migrate from the yolk sac wall to the gonads, where they give rise to the ovaries, with primordial follicles appearing around the 10th week. By the 12th week, stimulation by gonadotropins (Gn), luteinizing hormone (LH), and follicle‐stimulating hormone prompts the transformation of primordial follicles into primary and secondary oocytes surrounded by granulosa cells [107]. Ovarian development essentially halts before birth, with the ovaries only becoming responsive to gonadotropins during adolescence, leading to ovarian growth and oocyte activation. Ovulation capability is considered a fundamental indicator of maturation [108]. Additionally, the female reproductive tract originates from the differentiation of the Müllerian ducts (MD). Fusion of the MD medially results in the formation of the uterovaginal canal, with the cranial part developing into the uterus at 9‐10 weeks. By 16 weeks, the caudal portion of the uterovaginal canal beg–ins to exhibit characteristics of the cervix, with cervical glands emerging by 18 weeks [107].

Subsequently, postnatally, the growth of the uterus and oviduct decelerates, necessitating ongoing development and maturation under the influence of ovarian hormone stimulation during adolescence until the endometrium can undergo regular shedding in synchronization with the ovulatory cycle, culminating in the establishment of menstruation, signifying the fundamental maturation of the reproductive system. Recent research has indicated the involvement of RNA methylation in the developmental processes of the female reproductive system [109, 110]. Investigations have revealed that a decrease in demethylases (FTO and ALKBH5) and an increase in methyltransferases (METTL3 and METTL14) result in elevated m^6^A levels during gonadal development from 12.5 days post‐coitum (dpc) and 7 postpartum (pp) to adulthood. Furthermore, the m^6^A content was observed to be higher during the luteal phase compared to the follicular phase [111]. Currently, the precise mechanism through which m^6^A contributes to gonadal development remains incompletely understood.

As a reproductive organ, the ovaries exhibit accelerated aging compared to other organs [112, 113]. Relatively, the reproductive functions of the uterus and fallopian tubes may also be impacted by age, albeit less conspicuously than the ovaries [114], and are more susceptible to the effects of ovarian function decline and hormonal changes. Physiological ovarian aging, known as normal ovarian aging (NOA), is characterized by the gradual decline in the quantity and quality of oocytes with advancing age. Ovarian aging accelerates around 38 years of age, with the follicle reserve diminishing from approximately 250,000‐500,000 in newborns to fewer than 100–1000 at menopause [115]. Research has highlighted epigenetic regulation as a pivotal factor in the development and aging processes of the female reproductive system [116], with m^6^A modification likely playing a significant role [117]. However, the expression profiles of m^6^A methyltransferases and readers during ovarian aging necessitate further elucidation. Additionally, the implications of m^6^A levels and modifications in its regulatory elements, as well as strategies to enhance ovarian development, remain to be comprehensively understood. Hence, there is a need for further exploration into the significance of m^6^A and its regulatory components in governing the ovarian life cycle.

### 4.2 m^6^A Modification in Oocyte Development and Maturation

Follicle development progresses in tandem with oocyte maturation to establish a suitable microenvironment for oocyte viability. Normal follicle development and oocyte maturation are essential prerequisites for female fertility [118]. Oogenesis comprises three key stages: growth, maturation, and ovulation. Oocyte maturation involves the process wherein oocytes within the dominant follicle resume meiosis shortly before ovulation, transitioning from the diplotene stage of meiosis I to metaphase II (MII) [119]. This maturation process is often accompanied by RNA storage, translation, and degradation mechanisms to maintain transcriptional homeostasis in the transcriptome [120]. Maternal mRNA activation occurs in early oocytes and ceases at the germinal vesicle stage. Precise regulation of intracellular RNA posttranscriptional levels is crucial during oocyte development and maturation [121]. Oocyte maturation triggers a wave of RNA degradation, leading to the active degradation of approximately 20% of total maternal RNA [122]. Qi et al. observed that highly methylated mRNA is predominantly enriched during oocyte maturation and cell cycle progression mediated by progesterone, while m^6^A‐modified mRNA is primarily associated with biological processes such as transcription, protein phosphorylation, and cell division. This suggests that m^6^A modification plays a role in regulating RNA translation during oocyte maturation [123].

Ivanova et al. demonstrated the essential role of YTHDF2 in the posttranscriptional regulation of the maternal MII transcriptome, a critical determinant of oocyte competence crucial for early embryonic development [122]. YTHDC1 influences postnatal oocyte maturation, as elucidated by Kasowitz et al. who revealed that the nuclear m^6^A reader YTHDC1 governs alternative polyadenylation and splicing processes during mouse oocyte development. YTHDC1 is closely associated with pre‐mRNA 3′ end processing factors SRSF3, SRSF7, and CPSF6, deficiencies of which may alter the length of the mRNA 3′ non‐coding region in oocytes, impacting oocyte maturation (Figure 2) [124]. In 2018, Xia et al. generated a METTL3‐deficient zebrafish mutant, demonstrating that oocyte maturation and development were impeded, suggesting that METTL3 deficiency contributes to the cessation of oocyte maturation [125]. To support this hypothesis, researchers overexpressed METTL3 in the mutant, successfully rescuing oocyte maturation [125, 126]. Due to ethical and logistical constraints related to human oocytes, direct research on m^6^A modification‐related gene interventions in human oocytes is limited. Follicular fluid composition can serve as an indicator of oocyte quality. With advancing female age, ovarian reserve function and oocyte quality decline progressively. Comparative analysis of FTO expression in follicular fluid and m^6^A content across different age groups revealed a decrease in FTO expression with age, accompanied by an increase in m^6^A content, underscoring the intimate relationship between m^6^A and the maturation of human oocytes [127].

### 4.3 m^6^A Modification in Early Embryonic Development and Implantation

Embryonic development encompasses the progression from zygote formation to embryo detachment from the egg membrane, culminating in implantation on the endometrium. This intricate and sequential process relies on the precise interaction between gene selection and specific spatiotemporal expression, giving rise to a meticulously regulated network of gene products. Disruptions in gene expression can result in aberrant embryonic development, potentially leading to congenital malformations. Hence, investigating the regulatory role of m^6^A modification during embryonic development holds significant importance.

During early embryonic development and implantation, maternal‐to‐zygotic transition (MZT) is a genetic necessity involving the degradation of maternal RNA and DNA stores and the activation of the zygotic genome (ZGA) [128]. The critical biological process of early embryonic development is typically characterized by a programmed transition to the totipotent and pluripotent embryonic state, followed by cell fate determination and lineage‐specific differentiation [129]. MZT initiates upon the resumption of oocyte meiosis rather than fertilization. The targeted degradation of maternal mRNA sets the stage for zygotic genome activation and early embryonic development [130, 131]. Changes in mRNA stability induced by m^6^A modification play a crucial role in regulating MZT.

With an impressive 87% genetic similarity between zebrafish and humans, the zebrafish has been extensively utilized as a model organism in studies investigating m^6^A modifications in oocyte maturation and embryonic development. Research has revealed that over one‐third of maternal mRNA in zebrafish undergo m^6^A modification, with the clearance of these transcripts facilitated by YTHDF2. Defects in YTHDF2 have been shown to hinder maternal mRNA decay and impede zygotic genome activation, leading to a failure in MZT [132]. However, contrasting findings suggest that YTHDF2‐mediated m^6^A modification may not be essential during the early embryonic stage in zebrafish, as single knockout of YTHDF2 has minimal impact on maternal mRNA clearance or embryonic development [133]. Discrepancies in these conclusions may be attributed to variations in genetic backgrounds between mutant and wild‐type controls.

Further evidence of m^6^A involvement in regulating ZGA has been documented in mammals. Studies have shown that targeted mutagenesis of YTHDF2 disrupts the maternal function responsible for regulating the abundance of MII oocyte transcripts, subsequently impacting early zygotic development in mice [122]. YTHDF2 has also been identified as a crucial player in goat early embryogenesis by facilitating the clearance of maternal mRNA, potentially through the recruitment of deadenylases and mRNA decapping enzymes [134]. In mice, in addition to its role in promoting the degradation of maternal mRNA, the knockout of METTL3 results in decreased translation efficiency of maternal mRNA in oocytes, leading to impaired oocyte maturation and disruption of maternal mRNA degradation, thereby impeding the MZT process [122]. Maternal deletion of IGF2BP2 in mice has been linked to early embryonic arrest at the two‐cell stage [135]. The expression patterns of key regulatory factors during human MZT exhibit similarities to those observed in mice, although a comprehensive understanding of the kinetics and biochemical mechanisms involved in m^6^A reader proteins and RNA degradation remains incomplete [136].

Following fertilization in the fallopian tube, the egg initiates early embryonic development and differentiation. The zygote progresses into a blastocyst, which subsequently undergoes implantation into the endometrium, a process known as implantation. The outer layer of the blastocyst comprises trophoblast stem cells, which differentiate into the embryolemma and placenta [137]. The placenta plays a pivotal role in the pregnancy process, with placental abnormalities being closely associated with early embryonic demise and developmental anomalies. In placental trophoblasts of patients experiencing spontaneous abortion, a significant decrease in the demethylase FTO and leukocyte antigen HLA‐G levels has been observed, along with reduced expression of the target mRNA of YTHDF2. This suggests that aberrant methylation induced by FTO in chorionic cells alters immune tolerance and angiogenesis at the maternal‐fetal interface, ultimately leading to abortion [138]. Moreover, a notable reduction in the overall m^6^A modification level of mRNA in chorionic cells from patients with recurrent spontaneous abortion has been reported. Knockdown of ALKBH5 has been shown to decrease CYR61 mRNA stability, thereby inhibiting trophoblast cell proliferation and invasion at the maternal‐fetal interface in early pregnancy [139]. Dysregulation of m^6^A modification may disrupt trophoblast differentiation and pregnancy function, potentially resulting in compromised fertility, including abortion and pre‐eclampsia (PE). Trophoblast cells share similarities with tumor cells, such as invasion, angiogenesis, and an immunosuppressive environment, both relying on support from the immune microenvironment. Exploring m^6^A modification in tumor immune cells may offer insights into the dysfunction of stromal cells, trophoblasts, and decidua immune cells at the maternal‐fetal interface, shedding light on the underlying causes and molecular mechanisms.

## 5 m^6^A in Female Reproductive Diseases

Epigenetic regulation holds profound implications for female reproductive health. Extensive evidence highlights the pivotal role of m^6^A modification in physiological and pathological processes within the female reproductive system, intricately linked to a range of reproductive disorders [140, 141]. Unraveling the mechanisms underpinning these processes is paramount for identifying therapeutic targets for diseases and safeguarding women's reproductive health.

### 5.1 m^6^A Modification in Polycystic Ovary Syndrome (PCOS)

PCOS, a multifaceted endocrine disorder characterized by hyperandrogenism, ovulatory dysfunction, and polycystic ovaries, has been linked to m^6^A modifications in its pathophysiology [142, 143]. PCOS affects around 6%–10% of women of reproductive age and stands as a prominent cause of infertility [142]. Additionally, the syndrome is associated with insulin resistance, obesity, and an elevated risk of developing type 2 diabetes and cardiovascular diseases. Recent investigations have delved into the interplay between m^6^A RNA modification and PCOS, aiming to elucidate the potential mechanisms through which m^6^A may impact the onset and progression of the syndrome [144, 145].

FTO is a gene recently identified as being associated with obesity. Numerous studies have explored the link between FTO polymorphisms and PCOS, yielding inconsistent results likely influenced by population heterogeneity, sample size variations, and methodological differences. In a meta‐analysis conducted by Liu et al. the rs9939609 A/T polymorphism in the FTO gene was found to be linked to PCOS susceptibility, with the A allele posing a risk factor for the syndrome [146]. However, the precise relationship among PCOS, obesity, and FTO polymorphism remains ambiguous. Expanding on prior investigations, researchers speculate that the rs9939609 polymorphism in the FTO gene may heighten the predisposition to PCOS development [147]. Moreover, a study revealed that FTO overexpression reduced the m^6^A modification of FLOT2 transcripts, leading to increased expression in granulosa cells and consequent dysfunction [148]. While further comprehensive research is warranted to fully grasp the interplay between FTO and PCOS, FTO holds promise as a pivotal focal point for advancing our understanding of PCOS and as a potential target for therapeutic interventions.

Research indicates a significant increase in m^6^A levels in luteinized granulosa cells of PCOS patients, while the m^6^A modification of FOXO3 mRNA is decreased [149]. Notably, the targeted knockout of m^6^A methyltransferase or demethylase alters FOXO3 expression levels solely in the control group, not in the PCOS group. These findings suggest that aberrant m^6^A‐mediated regulation of FOXO3 posttranscriptional expression occurs in individuals with PCOS. FOXO protein plays a pivotal role in the insulin signaling pathway, hinting at a potential link between m^6^A modification and insulin resistance in the pathogenesis of PCOS. Additionally, Zhou et al.'s study revealed upregulated expression of FTO and Flotillin2 in granulosa cells of PCOS patients. Flotillin 2, a downstream target of FTO, is upregulated by FTO overexpression, leading to reduced insulin‐induced membrane transport of glucose transporter 4 (GLUT4) and subsequent induction of insulin resistance and dysfunction in ovarian granulosa cells [148]. In essence, m^6^A modification is linked to two key symptoms of PCOS, namely obesity and insulin resistance, aiding in a more comprehensive understanding of this reproductive endocrine metabolic disorder and offering clinical insights for PCOS patients grappling with infertility.

### 5.2 m^6^A Modification in Endometriosis

Endometriosis (EMS), also known as endometrial ectopia, refers to the presence, proliferation, and infiltration of endometrial tissue (glands and stroma) outside the uterine cavity and uterus, resulting in recurrent bleeding, pain, infertility, and the development of nodules or masses [150]. Prior research has indicated that aberrant gene methylation can induce abnormal gene expression, thereby fostering ectopic endometrial growth and altering the uterine milieu [151]. Li et al. [152] unveiled the pivotal role of the METTL3/m^6^A/miR‐126 pathway in the migration and invasion of endometrial stromal cells in EMS. Specifically, METTL3 downregulation diminishes the m^6^A modification level of pri‐miR‐126, impairing pri‐miR‐126 maturation into miR‐126 and consequently enhancing endometrial stromal cell migration and invasion. Similarly, Wang et al. [153] illustrated the critical involvement of the FTO/autophagy‐related protein 5 (ATG5)/recombinant pyruvate kinase isozymes M2 (PKM2) pathway in endometriosis. Their findings indicated that FTO suppresses ATG5 expression in an m^6^A‐dependent manner while elevating PKM2 expression, thereby boosting cellular glycolysis levels. Targeting the ATG5/PKM2 metabolic pathway via FTO was shown to inhibit glycolysis, proliferation, and metastasis of endometrial stromal cells. Studies have highlighted a significant downregulation of most m^6^A modification regulatory factors in ectopic samples compared to normal and in situ endometrium. Notably, HNRNPA2B1 and HNRNPC are differentially expressed m^6^A modification regulatory factors, potentially serving as m^6^A‐related diagnostic biomarkers for endometriosis [154]. Furthermore, observations in individuals with uterine muscle layer functional disorders have revealed distinct expression patterns of m^6^A regulatory factors compared to normal tissues [155]. The target genes of these factors may impact immune response and cell adhesion. This research underscores the role of m^6^A modification in modulating specific signaling pathways and underscores the significant value of m^6^A modification regulatory factors as prognostic markers and therapeutic targets.

### 5.3 m^6^A Modification in PE

m^6^A modification plays a pivotal role in the pathogenesis of PE, a distinct pregnancy‐related hypertensive disorder posing substantial risks to both the expectant mother and the fetus. Globally, approximately 2%–8% of pregnancies are affected by PE, characterized by the onset of hypertension and proteinuria after 20 weeks of gestation. The intricate pathophysiology of PE, involving impaired placental development, oxidative stress, inflammation, and endothelial dysfunction, remains incompletely understood. Recent evidence suggests that m^6^A modifications may contribute to the molecular mechanisms underlying PE. A study revealed that METTL3 induced ferroptosis, inhibiting trophoblast migration and invasion and in vivo PE symptoms by catalyzing the m^6^A modification of ACSL4 mRNA [156]. Moreover, research has indicated that endoplasmic reticulum (ER) stress‐induced dysfunction in nourishing cells, associated with METTL3‐mediated m^6^A modification, contributes to PE [157]. The m^6^A demethylase FTO, expressed in the placenta, exhibits altered expression levels in PE. Given FTO's role in regulating m^6^A status of transcripts involved in lipid metabolism, energy homeostasis, and oxidative stress response, changes in FTO activity could impact the metabolic and oxidative stress profiles observed in preeclamptic placentas [158]. Additionally, researchers observed significantly upregulated expression of demethylase ALKBH5 in the placenta of PE patients, with inhibition of its expression alleviating PE symptoms in pregnant mice. Downregulation of ALKBH5 expression increased the m^6^A modification level of PPAR‐gamma mRNA, leading to its upregulation. Subsequently, PPAR‐gamma activated the Wnt/β‐catenin pathway by enhancing histone methylation in the ALCAM promoter region, ultimately decelerating PE progression [159]. Despite the mounting evidence linking m^6^A modification to PE, numerous questions remain unanswered. Elucidating the precise molecular mechanisms through which m^6^A modulates specific genes and pathways in the context of PE is imperative. Moreover, exploring the potential of m^6^A‐targeted therapies for preventing or treating PE represents a promising yet largely unexplored research direction. Further investigations into the role of m^6^A in this intricate disorder may offer novel insights into its molecular foundations and open avenues for innovative therapeutic approaches.

### 5.4 m^6^A Modification in Female Reproductive System Neoplasms

m^6^A modification has been established to play a significant role in the initiation and progression of various tumors in the female reproductive system, including ovarian cancer, endometrial cancer (EC), and cervical cancer. Tumor development in the reproductive system can profoundly impact fertility, leading to conditions such as infertility, miscarriage, and preterm birth. Analysis of The Cancer Genome Atlas (TCGA) and Genotype‐Tissue Expression (GTEx) datasets through bioinformatics reveals distinct expression profiles of m^6^A modification‐related protein factors in cancerous tissues compared to normal tissues [29]. It is noteworthy that the functions of m^6^A regulatory factors exhibit variability across different tumors or within the same tumor under varying conditions, potentially either promoting or inhibiting cancer progression. The dual nature of m^6^A modification suggests a complexity in its regulatory mechanism beyond initial expectations, influenced by the cellular microenvironment.

Ovarian cancer is ranked as the third most fatal cancer among women globally within the spectrum of reproductive system malignancies [160]. The 5‐year survival rate for ovarian cancer patients remains relatively low due to the absence of early diagnostic methods and effective treatment strategies. In recent years, m^6^A modification, a significant epigenetic alteration, has emerged as a focal point for its involvement in ovarian cancer. Studies have revealed substantial differences in m^6^A modification levels and the expression of regulatory factors between ovarian cancer tissues and normal ovarian tissues. Specifically, the expression of METTL3 and METTL14 is typically elevated in ovarian cancer tissues, while FTO expression is comparatively reduced [161, 162, 163]. These alterations impact the overall m^6^A modification level, subsequently influencing the proliferation, invasion, and chemosensitivity of ovarian cancer cells. Notably, the regulatory interplay between YTHDF2 and miR‐145 in epithelial ovarian cancer, through a double‐negative feedback loop affecting m^6^A modification levels, offers novel insights into carcinogenesis and potential therapeutic targets [164]. Hypoxia, a pivotal factor in the tumor microenvironment, influences the expression of numerous genes through the activation and transcription of hypoxia‐inducible factor 1‐alpha (HIF‐1α). Lyu et al. demonstrated that in ovarian cancer cells, HIF‐1α regulates WTAP expression, which in turn interacts with DGCR8 to modulate miR‐200 expression via m^6^A modification, ultimately impacting aerobic glycolysis in tumor cells by upregulating the key glycolytic enzyme HK2 [165]. Progress in research on m^6^A modification in the onset and progression of ovarian cancer is rapidly evolving, presenting new targets for inhibiting the proliferation, invasion, and migration of ovarian cancer cells. However, challenges persist in addressing chemotherapy resistance and recurrence, hindering improvements in ovarian cancer prognosis. Hao et al. proposed that TRIM29 may function as an oncogene to promote chemotherapy resistance in a m^6^A‐YTHDF1‐dependent manner [166]. Additionally, Nie et al. suggested that sustained upregulation of the ALKBH5‐HOXA10 loop activates the JAK2/STAT3 signaling pathway, fostering platinum resistance in ovarian cancer [167]. Exploring these research avenues may offer insights into combating chemotherapy resistance and recurrence. In conclusion, researchers have identified numerous potential therapeutic targets for ovarian cancer treatment and elucidated drug resistance mechanisms, paving the way for novel treatment directions. However, identifying optimal treatment targets and strategies may present a new challenge for researchers.

According to a 2019 survey by the American Cancer Society, EC is identified as the most prevalent gynecological malignancy in developed countries, with 57% of cases associated with obesity. The increasing incidence of obesity, driven by lifestyle changes, is leading to a younger age of onset and a growing trend in EC, significantly impacting the physical and mental health of women [168]. Obesity is recognized as a risk factor for EC, as the accumulation of fat cells can result in heightened estrogen production. Estrogen, in turn, can regulate FTO expression through the activation of the phosphatidylinositol 3‐kinase (PI3K)/protein kinase B (AKT) and mitogen‐activated protein kinase (MAPK) signaling pathways, thereby promoting the proliferation and invasion of EC cells [169]. Furthermore, a study conducted by Zhang et al. [170] revealed significantly elevated levels of FTO and the oncogene HOXB13 in EC cells compared to normal tissue cells. Subsequent investigations unveiled that FTO catalyzes the demethylation of m^6^A in HOXB13 mRNA, inhibiting the YTHDF2‐mediated degradation of HOXB13 mRNA, thereby enhancing HOXB13 expression and activating the WNT signaling pathway, ultimately accelerating EC invasion and metastasis. This study further underscores the association between obesity and EC, emphasizing the potential of utilizing FTO as a therapeutic target for EC.

Cervical cancer stands out as the most prevalent malignant tumor in women, with multiple infections of human papillomavirus (HPV) posing a high‐risk factor for its development [171]. Hu et al. [172] initiated their study with HPV and discovered that silencing the E6/E7 genes leads to a reduction in the expression of IGF2BP2, consequently weakening the aerobic glycolysis capacity and growth of cervical cancer cells. Given that MYC is a downstream target of IGF2BP2 and a crucial regulator of glycolysis, further investigations revealed that E6/E7 can modulate aerobic glycolysis in cervical cancer cells through the m^6^A‐MYC pathway mediated by IGF2BP2 both in vitro and in vivo. The aerobic glycolysis pathway serves as the primary metabolic route for energy metabolism in tumor cells, characterized by a high glucose uptake rate, active glycolysis, and elevated lactate content in metabolic byproducts, commonly known as the Warburg effect [173]. Among the key enzymes involved in the Warburg effect, HK2 plays a significant role. Wang et al. observed that the METTL3/YTHDF1 complex boosts the stability of HK2 mRNA and elevates its protein expression in an m^6^A‐dependent manner, thereby fostering the Warburg effect [174]. Similarly to EC, there exists a potential m^6^A‐ncRNA regulatory mechanism in cervical cancer. Gong et al. demonstrated, through transcriptome sequencing analysis, that miR‐30c‐5p promotes ferroptosis in cervical cancer, inhibiting the growth and metastasis of cervical cancer xenografts by targeting the METTL3/KRAS axis [175]. PIWI‐interacting RNA (piRNA) represents a type of noncoding RNA that interacts with Piwi proteins. Dysregulated expression of piRNAs is closely linked to various cancers. In the context of cervical cancer, Xie et al. [176] illustrated that piRNA‐14633 serves as an upstream gene of METTL14. piRNA‐14633 stimulates the expression of CYP1B1 through METTL14‐mediated m^6^A methylation, thereby enhancing the proliferation, migration, and invasion capabilities of cervical cancer cells.

## 6 Detection and Identification of m^6^A Modification

The discovery of m^6^A methylation dates back to the 1970s. However, due to the constraints of sequencing technology and biochemical analysis methods during that period, the detection and identification of m^6^A modifications on a large scale were not feasible. Only a limited number of modified sites were identified in viral and cellular RNA. In recent years, the advent and advancement of high‐throughput sequencing technology have enabled the rapid and effective detection of m^6^A modifications on a broader scale. Leveraging m^6^A sequencing technology, researchers can now investigate epigenetic regulatory signals changes more efficiently. In the realm of ovarian aging and reproductive system diseases research, m^6^A sequencing has emerged as the preferred approach for screening m^6^A protein targets, significantly enhancing our comprehension of the functional role of m^6^A methylation in reproductive system development and aging. This has led to breakthroughs in understanding the link between modification irregularities and clinical diseases [177]. The introduction and concurrent utilization of novel m^6^A sequencing and identification technologies offer advantages such as reducing the need for scarce samples, mitigating batch effects, and enhancing the precise identification of modified sites and quantification of modification levels. This aids in elucidating upstream and downstream regulatory mechanisms [25] and facilitates the translation of fundamental research discoveries into clinical therapeutic applications. Presently, various m^6^A detection technologies present distinct pros and cons concerning cost, resolution, quantitative accuracy, and sensitivity. Researchers are advised to select the appropriate detection method based on their specific requirements (Table 2).

**Table 2:** Detection methods of m^6^A.

Techiques	Data	RNA types	Technological means	Resolution	Elapsed time	Quantification	Sensitivity	References
MeRIP‐seq	2012	mRNA/lncRNA	m^6^A antibody precipitation and high‐throughput sequencing	100–200 bp	Long	No	Medium	[34]
m^6^A‐seq	2012	mRNA/lncRNA	m^6^A antibody precipitation and high‐throughput sequencing	100–200 bp	Long	No	Medium	[35]
m^6^A‐LAIC‐seq	2016	PolyA RNA	m^6^A antibody, ERCC RNA Spike‐In Mix, and high‐throughput sequencing	Gene level	Long	Yes	High	[36]
PA‐m^6^A‐seq	2015	PolyA RNA	m^6^A antibody, UV365, 4SU, and high‐throughput sequencing	25–30 nt	Medium	Yes	High	[37]
miCLIP	2019	PolyA RNA	m^6^A antibody, UV254, and high‐throughput sequencing	1 bp	Long	No	High	[38]
m^6^A‐CLIP	2015	PolyA RNA	m^6^A antibody, UV254, and high‐throughput sequencing	1 bp	Long	No	High	[39]
m^6^ACE	2019	PolyA RNA	UV254, exonuclease XRN1, and high‐throughput sequencing	1 bp	Medium	Yes	High	[40]
m^6^A‐REF‐seq	2019	PolyA RNA	MazF/FTO degradation, and high‐throughput sequencing	1 bp	Medium	Yes	High	[40]
MAZTER‐seq	2020	PolyA RNA	MazF/FTO degradation, and high‐throughput sequencing	1 bp	Medium	Yes	High	[41]
DART‐seq	2019	PolyA RNA	APOBEC1‐ YTH protein, and high‐throughput sequencing	10 bp	Long	No	Low	[42]
scDART‐seq	2022	PolyA RNA	APOBEC1‐ YTH protein, single‐cell detection, and high‐throughput sequencing	10 bp	Long	No	Low	[43, 44]
m^6^A‐label‐seq	2020	mRNA	a^6^A, and high‐throughput sequencing	1 bp	Medium	Yes	High	[45]
m^6^A‐SAC‐seq	2022	polyA or rRNA‐depleted RNA	m6A‐selective allyl chemical labeling and high‐throughput sequencing	1 bp	Medium	Yes	High	[46]

### 6.1 MeRIP‐Seq and m^6^A‐Seq

The majority of RNA modifications exist at low abundance levels, and detecting chemical modifications directly on RNA is challenging due to the instability and intricate structure of single‐stranded RNA. During the construction of a cDNA library through the reverse transcription of single‐stranded RNA into double‐stranded cDNA, the inability to discern the methylation status of the adenosine N6 position results in the loss of information related to m^6^A modifications in the sequenced fragments. Therefore, a critical aspect of m^6^A modification detection involves distinguishing between modified and unmodified RNA fragments. This necessity has spurred the development of several antibody‐based high‐throughput detection techniques.

In 2012, MeRIP‐seq and m^6^A‐seq were the initial high‐throughput sequencing techniques independently developed by two distinct research groups [34, 35]. These methodologies were employed to map m^6^A modifications on mRNA, unveiling the landscape of m^6^A modifications and pinpointing over 7000 m^6^A modification sites within the human transcriptome. Presently, both approaches remain the principal sequencing methods for detecting m^6^A modifications. MeRIP‐seq/m^6^A‐seq entails the generation of two libraries: an immunoprecipitation (IP) library and an RNA‐seq library (Input). The Input library is constructed using conventional procedures, while the IP library involves the IP of m^6^A‐modified RNA fragments utilizing specific antibodies. In proximity to the m^6^A modification sites, there is an elevated frequency of read occurrence, resulting in a peak approximately double the read length. This characteristic facilitates the detection of methylated transcripts and the precise localization of m^6^A modification sites. MeRIP‐seq/m^6^A‐seq adheres to a standardized workflow and has been extensively employed as a pioneering technique for exploring the implications of m^6^A modifications in growth, development, diseases, and cancer research [132, 178, 179].

To enhance the detection precision of m^6^A modification sites, miCLIP and m^6^A‐CLIP (m^6^A individual‐nucleotide‐resolution cross‐linking and IP) techniques have been devised [39, 180]. Initially, purified RNA is fragmented and co‐precipitated with antibodies, followed by crosslinking under 254 nm ultraviolet light. Subsequently, the m^6^A antibody‐RNA complex is covalently linked to denaturing polyacrylamide gel electrophoresis and nitrocellulose (NC) membrane transfer. Uncrosslinked RNA fragments are not retained on the membrane, thereby reducing background noise. The liberated RNA fragments then undergo reverse transcription and high‐throughput sequencing. Despite the costliness and time‐intensive nature of these approaches, they effectively differentiate m^6^A from m^6^Am through the utilization of distinct antibodies and enhance the resolution of m^6^A identification to the single‐base level, rendering them valuable tools for m^6^A research.

Antibody‐based methods face challenges such as variability and limited resolution due to fluctuations in antibody affinity and batch effects. Additionally, many of the enhancement techniques for these methods require complex library construction processes. Therefore, a simple approach is needed that eliminates the need for antibody precipitation to determine the precise location of m^6^A modifications on nucleotides. MazF, a restriction endonuclease, has the ability to selectively recognize the ACA motif and cleave single‐stranded RNA at the 5′ side of the first A [181]. However, if the initial A contains an m^6^A modification (m^6^ACA), it hinders MazF's recognition. Through the integration of MazF and NGS technology, two separate research groups have introduced MAZTER‐seq [182] and m^6^A‐REF‐seq [183]. The sequencing process involves purified RNA being cleaved by MazF and then subjected to high‐throughput sequencing. The identification of m^6^A modification sites is determined by examining whether the cleavage sites of the restriction enzyme are internal or at the ends of the reads. With MazF's exceptional sensitivity and specificity, it can distinguish between m^6^A and m^6^Am. Moreover, the use of demethylase FTO‐treated mRNAs as negative controls can significantly reduce false‐positive results.

By leveraging the m^6^A metabolic pathway, the N6‐methyladenosine can be replaced with alternative modifications that are more easily detectable and isolatable, enabling convenient and efficient identification of m^6^A modifications. A prominent method for such approaches is the m^6^A‐label‐seq technique [42]. S‐adenosyl methionine (SAM) serves as a co‐factor for methyltransferases, which are responsible for methyl group transfer to adenine. In m^6^A‐label‐seq sequencing, allyl methionine is converted to allyl‐SAM or its selenium analog, allyl‐SeAM. This allyl‐substituted co‐factor produces N6‐allyl adenosine at the original m^6^A site, leading to the formation of cycloadduct N1‐N6‐cycloadduct adenosine (cyc‐A), causing base mismatches during reverse transcription. Through the analysis of the modified sites using bioinformatics, a high‐resolution map of m^6^A modifications at the single‐base level can be constructed.

With the advancement of genetic engineering, CRISPR gene editing technology is gaining popularity in the sequencing field. By combining cytosine deaminase (APOBEC1) with the YTH family of genes, APOBEC1 can be targeted to the m^6^A site using the YTH domain, enabling the conversion of the C in the RRACH motif to U. Subsequent NGS sequencing can identify the C‐to‐U conversion site occurring after the A, and DART‐seq [42]. is developed based on this principle for detecting m^6^A modification sites. Serving as a complementary method, it demonstrates efficacy in detecting alterations in m^6^A modification sites in biological samples.

### 6.2 Low‐Throughput Detection Techniques

High‐throughput techniques for detecting m^6^A modifications still face challenges, such as increased costs related to dual libraries, limited specificity of m^6^A antibodies that may cross‐react with sites like m^6^A and m, vulnerability of antibody binding sensitivity to experimental conditions, and insufficient resolution for accurate localization of modification sites. As a result, researchers have developed low‐throughput detection methods to reduce costs and improve the accuracy of identifying and quantifying m^6^A modifications.

The MeRIP‐qPCR assay is a suitable method for identifying m^6^A modifications on specific RNA molecules [59]. Initially, the enriched RNA‐antibody complexes are treated with proteinase to remove the antibody. Subsequently, the RNA undergoes reverse transcription polymerase chain reaction (RT‐PCR). This methodological enhancement enhances sensitivity, enabling the detection and semi‐quantification of m^6^A modifications even when working with limited samples. Typically, MeRIP‐seq techniques are used for comprehensive screening of m^6^A modifications, followed by validation using MeRIP‐qPCR to explore their regulatory roles on specific transcripts.

The m^6^A dot blot analysis is primarily utilized for semi‐quantitative assessment in m^6^A research. Samples containing RNA mixtures are spotted onto an NC membrane to create dots [184]. Following RNA immobilization and membrane drying, specific antibodies are used to probe the biological molecules. This method significantly reduces processing time by eliminating the need for complex procedures such as chromatography or gel electrophoresis. However, its application is limited to confirming the presence of m^6^A or comparing m^6^A levels among different experimental groups; it does not provide insights into m^6^A localization or precise quantification.

Due to the unique physical and chemical properties of m^6^A, its identification can be achieved through single nucleotide degradation combined with ultraviolet detection techniques. Liquid chromatography‐mass spectrometry (LC‐MS) has emerged as the predominant quantitative method for evaluating the overall m^6^A modification status of RNA [185]. Notably, RNase T1 and RNase A are the primary RNA degradation enzymes, breaking down RNA into small oligonucleotide fragments or individual nucleotides. By comparing individual nucleotides with their unmodified counterparts, the extent of m^6^A modification can be measured. While LC‐MS is easy to use and highly sensitive, it does not have the capability to precisely locate the m^6^A modification sites or identify the specific RNA molecules undergoing modification. Therefore, its usefulness is limited to capturing general changes in m^6^A modification levels.

## 7 Conclusions

m^6^A modification plays a crucial role in facilitating the cellular sorting and management of RNA, contributing to the organized metabolism and functional regulation of cells, thereby enhancing the precise control of gene expression. The female reproductive process is a complex physiological phenomenon that necessitates the coordinated interplay of multiple mechanisms and meticulous regulation. Undoubtedly, m^6^A modification significantly impacts the development and early embryonic stages of oocytes, highlighting the extensive potential of m^6^A modification in the realm of reproduction. Current research suggests that m^6^A modification is involved in diverse mechanisms within female reproductive physiological processes, governing the orderly RNA metabolism. Furthermore, disruptions in m^6^A modification are closely linked to diseases, with various signaling pathways regulating the pathological progression of these conditions.

Nevertheless, the molecular mechanisms governing the modification and regulation of m^6^A and its associated regulatory factors remain poorly understood, and there is a lack of in vivo experimental validation, significantly impeding its clinical translational potential. For example, the mechanisms through which m^6^A modulates early embryonic development and the functions of trophoblast cells and embryonic stem cells during the blastocyst stage are still unclear. Moreover, the influence of environmental factors on m^6^A regulation in embryonic development, such as exposure to the final metabolites of benzo(a)pyrene in cells or animals, and how it affects m^6^A levels, relevant methyltransferases, demethylases, and methyl‐binding proteins, along with their subsequent effects, remains to be elucidated. Furthermore, which other female reproductive disorders are associated with m^6^A, and what are the specific molecular mechanisms involved? Additionally, considering that m^6^A formation is a complex and dynamic process regulated by methyltransferases and demethylases, there is currently a lack of real‐time monitoring techniques for m^6^A, highlighting the need for advancements in existing detection technologies. A more profound comprehension of the regulatory mechanisms of m^6^A will enhance our understanding of the intrinsic processes of female reproductive system development and aging, offering more effective insights for disease treatment strategies and assisted reproduction.

## Author Contributions

Xiangrong Cui and Xuan Jing chose the subject and gave guidance for every step. Xia Huang, Tingting Xue, Huihui Li, Xinyu Zhu, and Shu Wang searched the literature and wrote the article. All authors read and approved the final manuscript.

## Ethics Statement

This review study was based on published work and therefore did not require approved by an institutional committee.

## Conflicts of Interest

The authors declare no conflicts of interest.

## Data Availability

The datasets used and/or analyzed during the current study are available from the corresponding author on reasonable request.
